# What evidence exists on the impacts of human activities on biodiversity and carbon capacity in North-East Atlantic blue carbon ecosystems: a systematic map protocol

**DOI:** 10.1186/s13750-025-00379-0

**Published:** 2025-12-08

**Authors:** Zina Kebir, Vera Helene Hausner, Ann Eileen Lennert, Amanda Poste, Carmen B. de los Santos

**Affiliations:** 1 Department of Arctic and Marine Biology, The Arctic University of Norway (UiT), Framstredet 39, Tromsø, 9019 Norway; 2 Department of Arctic Ecology, Norwegian Institute for Nature Research (NINA), Hjalmar Johansens gate 14, Tromsø, 9007 Norway; 3 Institute of Marine Sciences of Andalusia (ICMAN), Spanish National Research Council (CSIC), Puerto Real, Cádiz, 11519 Spain

**Keywords:** Coastal ecosystems, Blue carbon, Anthropogenic impacts, Biodiversity, Carbon sequestration, Organic carbon, Human activities

## Abstract

**Background:**

Coastal ecosystems, including seagrass meadows, saltmarshes, and macroalgae, are crucial in the sequestration and storage of organic carbon. These ecosystems provide essential ecosystem services, such as supporting biodiversity, coastal protection, and water quality enhancement. Despite their significance, they face substantial threats from human activities, including pollution, habitat degradation, and overexploitation, further exacerbated by climate change phenomena like heatwaves and ocean acidification. Efforts to protect, restore, or alleviate pressures on blue carbon ecosystems can yield multifaceted benefits beyond climate mitigation, including preserving biodiversity, enhancing climate resilience, and safeguarding vital services for human well-being. Understanding the factors affecting the biodiversity and carbon capacity i.e. the capacity for carbon uptake, storage and sequestration, of these ecosystems is crucial for effective conservation efforts. The goal of the present study is to assess the available quantitative and qualitative evidence on the impacts of human activities on the biodiversity and carbon storage capacity of blue carbon ecosystems in the North-East Atlantic. Developing a systematic map of the available evidence could significantly enhance our understanding of the pressures faced by blue carbon ecosystems in the North-East Atlantic and facilitate the identification of knowledge clusters and gaps thereby determining the scope and depth of the current knowledge base.

**Methods:**

A systematic map on existing evidence of human impacts on the biodiversity and carbon capacity of blue carbon ecosystems in the North-East Atlantic will be conducted using relevant bibliographic databases and a web-based search engine. All searches will be conducted in English and will gather peer reviewed publications from 1980 to 2024. The resulting literature will be screened by two independent screeners at the level of title and abstract followed by full text against a set of eligibility criteria (i.e. population, intervention, outcome, study type). Metadata will be extracted from studies that meet the eligibility criteria and summarize with heatmaps, bar plots, geographic distribution maps, and tabular summaries.

**Supplementary Information:**

The online version contains supplementary material available at 10.1186/s13750-025-00379-0.

## Background

In recent years, research has emphasized the global importance of coastal ecosystems - seagrass meadows, saltmarshes and macroalgae - to the sequestration and storage of organic carbon also referred to as blue carbon [2, 16, 18]. These blue carbon ecosystems deliver a wide range of valuable services while supporting high biodiversity, with many marine species and birds using them as nursing ground, shelter and food [4, 19, 23, 28]. In addition to carbon sequestration, they also provide protection against waves through stabilizing, accreting sediments and enhance water quality by filtering incoming nutrients such as nitrogen from surrounding watersheds [1, 2]. While these diverse functional roles and ecosystem services emphasize the important value of blue carbon ecosystems, these have been impacted by people for centuries due to land-derived pollution (e.g. micro-plastic, heavy metals, sewage discharge, etc.), degradation of natural habitats, overexploitation of coastal marine resources, alien species and other stressors deriving from increasing demand for space and resources from traditional and new industries [8, 12]. Once climate change effects such as heat waves and ocean acidification are added to direct human pressures, these ecosystems become some of the most threatened in the world [14, 22, 27, 29].

**Fig. 1:**
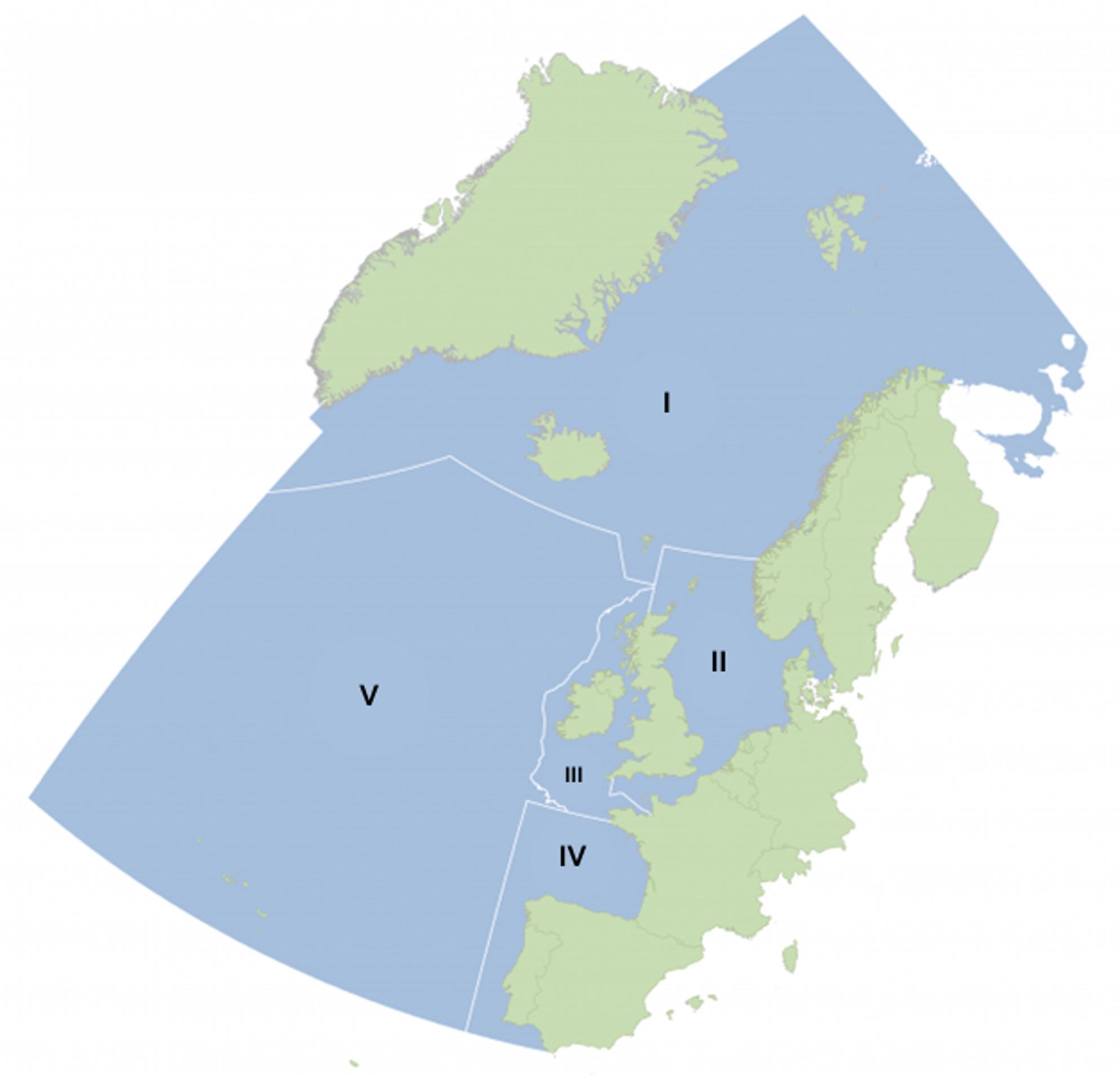
Map of the OSPAR regions. (Modified from: OSPAR Quality Status Report 2023; https://oap.ospar.org/)

This is particularly true for the North-East Atlantic which has a long history of intensive human use. The North-East Atlantic (Fig. 1) is bordered by 12 countries and its waters support a wide variety of human activities including commercial fishing and shipping, recreation and oil and gas industry [10, 11]. Likely because it is a heavily exploited area, it is also a well-studied and data rich area, monitored by multiple countries organized in OSPAR (Convention for the Protection of the Marine Environment of the North-East Atlantic) and ICES (International Council for the Exploitation of the Sea) [10, 26]. Despite being a well investigated area of the world, and even though anthropogenic pressures and their cumulative impacts on marine ecosystems have been subject of multiple research initiatives (e.g. [7, 13, 21], no previous studies have attempted to synthesize the knowledge about the combined impact of human activities on the capacity of the ecosystems to store carbon or protect biodiversity in the North-East Atlantic. A systematic map of the evidence available could identify knowledge clusters and gaps thereby determining the breadth and depth of the current knowledge base. This would enhance ecological knowledge and our understanding of the pressures that blue carbon ecosystems are facing and organize ecological information for decision-makers in the North-East Atlantic. Synthesizing and mapping a general state of scientific research on human impacts on blue carbon ecosystems represents a necessary step towards identifying targeted management and governance measures and actions aimed at protecting, restoring or reducing pressures on blue carbon ecosystems. The insights gained from this process are highly relevant not only for the North-East Atlantic but also for other regions with similar ecosystems and impacts, offering valuable lessons for global conservation efforts. Such efforts can provide multiple benefits beyond climate change mitigation, such as halting decline of biodiversity, enhancing climate resilience and safeguarding important services for human wellbeing especially for local coastal communities [2, 15].

### Stakeholder engagement

This systematic map was initiated by an international team of researchers with expertise in coastal ecology and blue carbon ecosystems. At this stage, no formal engagement with external stakeholders has taken place. However, the outcomes of this work are designed to be directly relevant to key regional and international stakeholders, particularly the OSPAR Commission, which coordinates marine environmental protection across the North-East Atlantic. The synthesis will also be of value to coastal managers, policymakers, conservation practitioners, and non-governmental organizations involved in marine habitat restoration, conservation planning, and climate mitigation in the North Atlantic region.

## Objective of the review

The objective of this systematic map is to identify, collate and map the current available quantitative and qualitative evidence on human impacts on biodiversity support and carbon capacity of blue carbon ecosystems in the North-East Atlantic region define by the OSPAR commission (Fig. 1). The OSPAR Maritime Area includes important migratory routes for seabirds, marine mammals and fish that are using the different ecosystems along the coasts for feeding, nursing and nesting. In terms of human impacts, the region faces many threats from e.g. pollution from land and shipping, coastal development and unsustainable fishing. Some countries in the region are at different stages of research on impacts of human activities on blue carbon ecosystems, and so the geographical scope will help catalog the evidence from several interconnected countries sharing the use of the North-East Atlantic Ocean. But also, by acknowledging that countries have different coastal development and pressures on the marine environment, this systematic map will provide localized information capturing location specific impacts as well as broader North-East Atlantic knowledge base. The lessons learned here and the results from this systematic map will additionally be shared with the OSPAR commission creating a space for discussion and feedback.

Our protocol was designed and tested in the OSPAR maritime area, but its main structure can also be used in other European seas or ecozones. To apply it in places such as the Mediterranean or Macaronesian regions, a few points should be considered. First, search terms and spatial limits may need to be changed to fit data sources. Second, habitat types and management systems can be different, so the protocol should be aligned with local classifications, such as EUNIS codes or the Barcelona Convention system. Third, metadata attributes (Table 4) may need adjustments regarding vegetation species or human interventions, yet it is not expectable that the outcome attributes may change. Making these adjustments will help the protocol stay clear and effective across different EU marine areas and support its use for policy and conservation goals beyond the OSPAR region.

We aim to synthesize this knowledge in a systematic map to provide structured and reliable information and be able to identify and point to important knowledge gaps, future research and management priorities. We use the term “human impacts” to mean the substantial environmental effect(s) from environmental state change caused by pressure(s) from human activities (Box 1).

This systematic map will seek to:

1. Identify research on human impacts on biodiversity and carbon capacity of blue carbon ecosystems in the North-East Atlantic region.
2. Identify the types of activities, pressures, and impacts assessed in the available studies.
3. Document, evaluate, and identify gaps that exist in the current research.
4. Make suggestions for future research priorities related to human impacts on coastal biodiversity and carbon capacity.

The central question to be addressed by the systematic map is: What quantitative and qualitative evidence exists regarding the impacts of human activities on the biodiversity and carbon capacity of blue carbon ecosystems in the North-East Atlantic? We will ask the following sub-questions to complete the analysis:

- How does the extent and distribution of evidence on human impacts on coastal ecosystems differ by ecosystem type (e.g., salt marsh, kelp), human activity type (e.g. trawling, aquaculture), and geographic location?
- What approaches (e.g. qualitative, quantitative, mixed method) are used to assess human impacts on biodiversity and/or carbon capacity?
- What metrics (e.g. species richness, species abundance, organic carbon sequestration rate) are used to assess biodiversity and/or carbon capacity?
- How does the extent and distribution of evidence on human impacts on blue carbon ecosystems differ between biodiversity and/or carbon capacity assessment?

Elements of the primary question: Elements of the primary question include the population, intervention, comparator, and outcome (Table 1).

**Table 1:** Summary elements of the primary question following ROSES (Reporting standards for systematic evidence Synthesis) nomenclature

PICO criteria	Elements from primary question
Population	Blue carbon ecosystems - seagrass meadows, saltmarshes and macroalgae in the North-East Atlantic
Intervention	Human activities in marine and coastal areas of the North-East Atlantic
Comparator	No comparator required
Outcome	There are no predefined outcomes. All observed effects related to biodiversity and/or carbon capacity of blue carbon ecosystems induced by pressures from human activities, including but not limited to changes in species diversity, habitat structure, biomass, carbon storage, productivity, etc.

##  Methods

This protocol is developed following the Collaboration for Environmental Evidence (CEE) Guidelines and Standards for Evidence Synthesis in Environmental Management [25] and will conform to the RepOrting standards for Systematic Evidence Syntheses in environmental research (ROSES) guidelines published in 2018 [6].

Box 1. Terminology *Carbon capacity* the ability of an ecosystem to absorb carbon dioxide (CO2) from the atmosphere (uptake), store carbon in biomass and sediments (carbon storage), sequester carbon over long periods (sequestration), and function as a net carbon sink.

*Carbon storage capacity* capacity of an ecosystem to store carbon in biomass and sediments.

*Carbon sequestration capacity* capacity of an ecosystem to store carbon in a carbon pool, i.e. a reservoir where carbon can reside in various chemical forms, for a long (several decades or centuries) period of time (IPCC, 2021; Nellemann et al., 2009; Trumper et al.,^ 2009^).

*Carbon sink capacity* any process, activity or mechanisms which allow an ecosystem to remove more CO2 from the atmosphere than it releases (IPCC, 2021; Pearson et al.,^ 2023^).

*Carbon uptake* a process by which the ecosystems absorb carbon from the atmosphere (Frigstad et al.,^ 2021^).

*Driver* a need arising from society, such as demand for food, energy, space, transport, security, or recreation, that triggers environmental changes in the state of the natural system through human activities.

*Human activity* activities operated by humans in the marine and coastal environment to answer the different needs of society (i.e. Finfish aquaculture is a human activity in relation to the need for food by cultivating living resources).

*Pressure* a result of a human activity causing an effect on any part of an ecosystem that can lead to changes in the state of the natural system (i.e. Finfish aquaculture (human activity) can lead to the loss of, or change to, natural biological communities through input of nutrients (pressure) or spread of non-indigenous species (pressure).

*State* the actual condition of the ecosystem and its components established in a certain area at a specific time frame, that can be quantitatively-qualitatively described based on physical (e.g. temperature, light), biological (e.g. genetic-, species-, community-, habitat-levels), and chemical (e.g. nitrogen level, atmospheric gas concentration) characteristics (source: [20].

*Impact* consequences of changes in ecosystem state on ecological characteristics (i.e. spread of non-indigenous species (pressure) from finfish aquaculture can disrupt ecosystem stability and food webs structure through competition leading to a decrease of native species (impact)).

*Biodiversity* The variability among living organisms from all sources including terrestrial, marine and other aquatic ecosystems and the ecological complexes of which they are a part. This includes variation in genetic, phenotypic, phylogenetic, and functional attributes, as well as changes in abundance and distribution over time and space within and among species, biological communities and ecosystems (source: [24].

## Searching for articles

### Search strategy

A comprehensive search will be performed to acquire peer-reviewed publications from 1980 to 2024 using Web of Science (WoS) and SCOPUS bibliographic databases in combination with a web-based search on Google Scholar. The year 1980 was selected as a starting point for this review because it closely follows the adoption of the EU Birds Directive in 1979 – the first major policy aimed at protecting coastal ecosystems in the North-East Atlantic. By addressing both terrestrial and marine environments, this directive marked an important moment in environmental conservation, making 1980 a meaningful baseline for assessing human impacts. While grey literature may provide additional information, materials found there are often less accessible, language-restricted, or primarily descriptive rather than analytical, and high-quality insights from this literature type are typically already incorporated into peer-reviewed publications. For these reasons, grey literature will not be included in this study. However, our strategy will also include hand-searching reference sections during the initial scoping from relevant peer-reviewed reviews previously published to identify potential additional publications that may not be found in our search.

### Language

The search will be conducted in English, including only studies with full texts available in English. Non-English studies with English titles and abstracts may be screened, but those lacking English full texts will be excluded and noted as non-English. We acknowledge that limiting to English may exclude relevant studies and introduce bias to our research.

### Geographical scope

The systematic map will include evidence from the North-East Atlantic region defined by the OSPAR commission. The OSPAR convention (Convention for the Protection of the Marine Environment of the North-East Atlantic) was signed in 1992 by the contracting parties to the original Oslo and Paris Conventions, which are Belgium, Denmark, Finland, France, Germany, Iceland, Ireland, the Netherlands, Norway, Portugal, Spain, Sweden and the United Kingdom of Great Britain and Northern Ireland along with Luxembourg, Switzerland and the European Union. OSPAR is the mechanism by which the governments of these countries and the European Union cooperate to protect the marine environment in the North-East Atlantic, providing a framework for cooperation to facilitate and coordinate the EU Marine Strategy Framework Directive, which aims to achieve a healthy, productive and biologically diverse North-East Atlantic. The OSPAR Maritime Area encompasses the whole of the North-East Atlantic Ocean, and it is divided into 5 regions (Fig. 1):

- *Region I* Arctic Waters – This region includes the Arctic Ocean and adjacent seas along the coast of East Greenland, Iceland, Norway and the Faroe Islands and is characterized by harsh climate, ice coverage and unique ecosystems. It has a low population density but some activities such as fishing, and oil production remain significant.
- *Region II* Greater North Sea - Covering the North Sea, including the English Channel, this region is one of the busiest maritime areas. It is highly industrialized with high maritime traffic. The countries in this region are Belgium, Denmark, France, Germany, the Netherlands, Norway, Sweden, and the United Kingdom.
- *Region III* Celtic Seas - This region encompasses the waters west of Scotland, Ireland, and the Celtic Sea. It is known for its wide variations in coastal topography including fjords bays, estuaries and sandy beaches and high biodiversity and fishing activities. The countries in this region are Ireland, the United Kingdom, and France.
- *Region IV* Bay of Biscay and Iberian Coast - Including the Bay of Biscay and waters along the Iberian Peninsula, this region features, bottom topography from continental shelf to abyssal plain and support rich fish fauna and important migration routes for sea birds. These features support fisheries and the tourism industry. The countries in this region are France, Spain, and Portugal.
- *Region V* Wider Atlantic - This region covers the open represents the deep waters of the North-East Atlantic where fragile deep-sea habitats and ecosystems have been recently discovered. Main human activities in this region are fishing and maritime transport.

### Keywords development

A scoping exercise of Web of Science (WoS) was conducted to build a relevant search string, using terms describing impacts of human activities on the blue carbon ecosystems analyzed in this study. A test list of 10 relevant scientific articles, which encompassed literature on blue carbon ecosystems in the North-East Atlantic was created by the authors to develop a comprehensive and robust search string (Additional file 3). Keywords and further relevant words were identified and extracted from the title and abstract of these papers using the R package ‘litsearchr’ [3]. Once the list of keywords was assessed complete by the review team, appropriate synonyms were identified using a thesaurus dictionary.

An example of this process for keyword development is as follows. Initial keywords related to the elements of the primary research question for human activities (intervention), blue carbon ecosystems (population) and impacts (outcomes) were developed by the reviewing team. A simple search string was created and applied in WoS to test the keywords and capture a focused set of relevant literature:

(TS= (“coastal habitat” OR “intertidal habitat” OR “subtidal habitat” OR “coastal ecosystem” OR “blue carbon” OR “blue carbon ecosystem” OR “blue carbon habitat” OR marsh* OR saltmarsh* OR estuar* OR kelp OR “macroalga” OR seaweed OR rockweed* OR seagrass* OR “sea grass” OR eelgrass OR “coastal wetland”).

AND.

TS=(impact* OR effect*) OR “human disturbanc*” OR pressure* OR “human impact*” OR “human pressure*” OR “human activit*” OR “anthropogenic impact*” OR “anthropogenic pressure*”)

AND.

TS=(biodivers* OR “biological diversity” OR “species richness” OR “species abundance” OR “species diversity” OR “macrofauna” OR biomass OR “carbon sequestrat*” OR “carbon stor*” OR “carbon stock*” OR “organic carbon”)))

AND.

AD=(“Azores” OR “Basque” OR “Belgium” OR “Danmark” OR “Danish” OR “Denmark” OR “Dutch” OR “English” OR “England” OR “Faroe Island*” OR “Faroe*"OR “France” OR “French” OR “Germany” OR “German” OR “Greenland” OR “Greenlandic” OR “Holland” OR “Iceland” OR “Icelandic” OR “Ireland” OR “Irish” OR “Netherland” OR “Norway” OR “Norwegian” OR “Portugal” OR “Portuguese” OR “Scotland” OR “Scottish” OR “Spain” OR “Spanish” OR “Sweden” Or “Swedish” OR “United Kingdom” OR “UK” OR “Wales”))

In this search, the (*) acts as a wildcard, which represents any character, so a string such as activit* would for instance represent activity and activities. Quotations marks are used to specify exact phrases. During the scoping exercise, relevant papers that omit to mention their study location in the title, abstract or keywords were identified. Therefore, the review team decided to limit the geographical scope of the search by investigating institution names and/or location from authors’ addresses in the Author Information, Address Field instead (“AD=” in WoS). Following this simple search, results were exported and run through the R package ‘litsearchr’, which use a quasi-automated method by text-mining and keyword co-occurrence to identify potential keywords [3].

### Search string development

Using the compiled keywords list, search strings were developed to align with the elements of the primary question representing population, interventions and outcomes. The population search string targeted eligible coastal ecosystems (i.e. seagrass, salt marsh, macroalgae) and also included more general terms, like marsh or seaweed, used to refer to these ecosystems (Table 2). The intervention strings included mostly general terms related to human activities and related pressures, while the outcomes string targeted terms related to biodiversity and carbon capacity increasing the likelihood to capture all relevant literature.

**Table 2:** Search substrings representing population, interventions, and outcomes elements (PIO criteria of the ROSES protocol)

PIO criteria	Concept	Substring (WoS syntax)
Population	Blue carbon ecosystems	(TS=(“coastal habitat” OR “intertidal habitat” OR “subtidal habitat” OR “coastal ecosystem” OR “blue carbon” OR “blue carbon ecosystem” OR “blue carbon habitat” OR marsh* OR saltmarsh* OR estuar* OR kelp OR “macroalga” OR seaweed OR rockweed* OR seagrass* OR “sea grass” OR eelgrass OR “coastal wetland”) AND AD=(“Azores” OR “Basque” OR “Belgium” OR “Danmark” OR “Danish” OR “Denmark” OR “Dutch” OR “English” OR “England” OR “Faroe Island*” OR “Faroe*"OR “France” OR “French” OR “Germany” OR “German” OR “Greenland” OR “Greenlandic” OR “Holland” OR “Iceland” OR “Icelandic” OR “Ireland” OR “Irish” OR “Netherland” OR “Norway” OR “Norwegian” OR “Portugal” OR “Portuguese” OR “Scotland” OR “Scottish” OR “Spain” OR “Spanish” OR “Sweden” Or “Swedish” OR “United Kingdom” OR “UK” OR “Wales”)
Intervention	Human activities and related pressures	(TS= (impact* OR effect OR effects OR “human disturbance*” OR “anthropogenic disturbance*” OR pressure* OR “human impact*” OR “human pressure*” OR “human activit*” OR “anthropogenic impact*” OR “anthropogenic pressure*”))
Outcome	Biodiversity	(TS= (biodivers* OR “biological diversity” OR “biotic diversity” OR “species richness” OR “species abundance” OR “eveness” OR “species diversity” OR “communit* structure” OR “species composition*” OR “ecosystem* integrity” OR “macrofauna”))
Outcome	Carbon capacity	(TS= (biomass OR “carbon sequestrat*” OR “carbon stor*” OR “carbon stock*” OR “organic carbon” OR “carbon uptake*” OR “carbon sink*” OR “carbon capture*”))

The population, intervention and outcomes search strings (Table 2) were used to capture certain types of articles following different combinations of search strings (Table 3). For example, we combined strings for populations and impacts of human activities on biodiversity or carbon capacity to search for articles where impacts of human activities affect either the biodiversity of blue carbon ecosystems or their carbon capacity. We then constructed a string combining human impacts on both biodiversity and carbon capacity to find articles where human impacts on blue carbon ecosystems have been used in reference to biodiversity and carbon capacity. The geographical scope was always added to the search string combination using “AD=”.

**Table 3:** Search string combinations

String combination	Search designed for
Impacts of human activities AND blue carbon ecosystems AND Biodiversity OR Carbon Capacity	Articles focused on impacts of human activities and targeted ecosystems and mention biodiversity or carbon capacity concepts in title, abstract or key words
Impacts of human activities AND blue carbon ecosystems AND Biodiversity AND carbon capacity	Articles focused on impact of human activities and targeted ecosystems and mention biodiversity and carbon capacity concepts in title, abstract or keywords

### Searching the literature

#### Bibliographic database

Searches will be carried out using the following databases:

- Web of Science Core Collection on the Web of Science platform (Clarivate) using the access rights provided from the Arctic University of Norway (UiT). The search covered the following indexes: SCI-EXPANDED, SSCI, CPCI-S, CPCI-SSH and ESCI.
- SCOPUS (Elsevier) using the access rights provided from the University of Algarve (UAlg) through the Centre of Marine Science of the Algarve (CCMAR).

#### Web-based search engine

Google Scholar will be used to capture additional relevant articles. The Boolean logic is limited in Google Scholar compared to platforms such as Web of Science, thus we will adapt our search string accordingly using only the most relevant components [5]. The search terms will be inserted in ‘With all the words’ box in ‘Advanced Search’ and we will perform the search on articles full text because title searches tend to return more grey literature [5]. We will screen the first 1000 search returns, based on recommendations for systematic searches in Google Scholar [5].

Web of science, Scopus and Google Scholar contains the mainstream research outlets on marine and coastal environments including all the aspects of the science but also the management and conservation of these environments, their organisms and resources and other economical and sociological aspects making them adequate to map research trends, knowledge gaps and clusters needed to fulfill the purpose of this review.

As the search strings were initially designed using Web of Science syntax, they will be adjusted as necessary to align with the specific syntax or limitations of other platforms. Any changes or adjustments made to search strings, as well as any applied filters or limiters, will be recorded in detail to ensure transparency and reproducibility.

### Search update

No search update will be undertaken as it is a short-term project and the original searches, and the production of the systematic map will take place during the same year.

### Comprehensiveness of the search

The comprehensiveness of the search attempt in WoS was tested against its ability to return the 10 relevant articles from the test-list (Additional file 3). These articles, which we refer to as benchmarking articles, met the eligibility criteria and would be included in the full text stage. We implemented our search string in WoS and used the field code ‘Topic’ which includes title, abstract and the article’s keywords. We adjusted our search string incrementally until the 10 articles indexed were captured by the search. Of the 10 articles in test-list, 10 were indexed in Web of Science Core Collection. The final search string will be used to search bibliographic databases and search engines for articles published over the period 1980–2024. Searches will be made for peer-reviewed articles.

### Article screening and study eligibility criteria

#### Screening process

Title and abstract of articles identified during the search process will be screened to assess whether they meet the predefined eligibility criteria. Screening at the title and abstract level will be conducted by two screeners using the web-based software application Swift Active Screener [9], which is a reference screening software designed for managing and analyzing data from systematic reviews. Swit Active Screener utilizes active learning, a machine learning approach, to prioritize publications by relevance, based on feedback from screeners, ensuring that relevant publications are reviewed earlier in the process. The software dynamically updates the ranking and relevance of publications as screening progresses. Additionally, it provides a real-time estimate of the percentage of relevant references screened from the initial dataset (referred to as the “recall rate”) and an estimate of the number of relevant references yet to be screened. These estimates enable users to set a target recall rate, at which point the screening process can be concluded. For our screening process, we selected a target recall rate of 95%, a percent comparable to human error rates [9]. Additionally, the software indicates whether there are conflicts in the inclusion and exclusion decisions made by the screeners. These conflicts will be resolved by discussions between the two screeners or consultation with a third party if consensus is not reached. This software has been shown to save significant time resources by using active learning and its associated ranking system. We acknowledge that the use of Swift Active Screener may introduce potential bias into our mapping results, as some articles ranked lower in relevance might be excluded despite warranting inclusion, potentially leading to their oversight. However, given that we expect over 5,000 articles and considering the analysis from Howard at al. 2020, we believe using Swift Active Software is necessary and worthwhile for this systematic map.

There will be two sequential levels of articles screening: a screening of the title and abstract followed by a screening of the full body text. Due to the anticipated high volume of articles, we will not perform a full double extraction at the full text stage. Instead, we will carry out consistency checks on a small subset of articles, as many as 5% of articles at the full text screening stages. The exact proportion of articles selected for these checks will be determined based on the total number of articles. The consistency checks will be performed using the Kappa statistics [17]. All studies excluded during full text screening stage will be kept in a record including the reasons for exclusion. At each stage of the screening process the articles will be compared to the eligibility criteria agreed on by the screeners. Data extraction will be conducted during the full-text screening stage according to the instruction in the data extraction codebook (Additional file 4).

Consideration of the potential limitations of this study will be integrated into the interpretation of the findings of this review. There is a possibility that the results of the searches differ from the study criteria due to the complexity of the question (i.e. numerous pressures and impacts), the lack of consistency of the synonyms for the keywords identified and the potential use of different definition of the terms “blue carbon ecosystems”, “biodiversity” and “carbon capacity”. Therefore, many non-relevant studies might be excluded. To overcome this, the screeners will try to identify the most representative key words and build a combination of main keywords with highly compatible additional keywords.

#### Eligibility criteria

To pass the screening process and be included in the map, articles need to meet the following eligibility criteria.

##### Relevant population(s)

This systematic map focuses on three types of coastal habitats: salt marsh, seagrass and macroalgae in coastal. The three ecosystems were selected because they qualify as blue carbon ecosystems meaning ecosystems that are important to the sequestration and storage of organic carbon [2, 16, 18]. The study focuses on the North-East Atlantic maritime area system defined as the OSPAR convention from the mid-Atlantic ridge in the west to the North Sea in the East and from the north Pole southward to the Azores (Fig. 1).

##### Relevant intervention(s)

Any type of human activities in coastal and marine areas in the North-East Atlantic driving changes in blue carbon ecosystems will be considered as relevant interventions (e.g., land-use, fishing, aquaculture, commercial shipping, industrial coastal development).

##### Relevant outcome(s)

This systematic map aims to determine the evidence base surrounding impacts of human activities on the biodiversity and carbon capacity of various coastal ecosystems. The study aims to describe and assess the past and current direct and indirect impacts of human activities and encompass therefore a wide range of potential outcomes. No predefined outcomes will be set as an outcome can be potentially relevant as long as related to the population studied, including any aspect related to impacts on biodiversity (e.g., change in food web interaction, change in species abundance) and/or carbon capacity (e.g., loss of organisms that promote carbon sequestration, change in above- and belowground carbon stocks, fluxes, and biomass).

##### Relevant study type(s)

Observational (e.g., monitoring, assessment), experimental and modeling studies will be included from the identified peer-reviewed articles. Theoretical studies, commentaries, perspectives, opinions, reviews, proceedings, thesis, book chapters and editorials will not be included.

### Study validity assessment

A critical appraisal of study validity will not be conducted, as the primary objective of the systematic map is not to evaluate the robustness of individual study designs but to compile a broad evidence base. However, information on study designs will be collected. This will enable future assessments of the validity of these methods if needed. We understand that not performing critical appraisals may have implications for the utility of the systematic map, such as limiting the interpretation of evidence gaps and clusters. These limitations will be acknowledged in the final map.

### Data coding strategy

For each article meeting the inclusion criteria, screeners will code the full text and extract relevant metadata attributes into a standardized data coding spreadsheet (Table 4) using Microsoft Excel. Several categories of data will be extracted (Table 4, Additional file 4). If information for a certain attribute is absent or not reported in the article, screeners will code the attribute as “unknown” or “unspecified”, as we do not intend to contact authors for missing information. Attributes not applicable to a particular article will be coded as “not applicable” (Additional file 4). A training session will be held to ensure consistency in data coding between screeners. Several articles representing straightforward to more difficult and nuanced articles will be selected. Each screener will receive the same subset of articles to code. Coding results, and inconsistencies will then be discussed and if needed, changes in attributes and instructions will be made.

**Table 4:** Metadata attributes to be extracted from articles selected after full text screening

Category	Attribute name	Description
General	Study ID	Unique identifier for each article
	Source	Bibliographic database name (WOS, SCOPUS)
	Screener	Screener name
	Date	Screening date (DD-MM-YYYY)
	Exclusion/inclusion	Record removed before full text screening
	Reason for Exclusion	Reason for removal
Bibliographic	Author(s)	Last name, Initial First Name
	Publication year	YYYY
	Title	Title of the study
	Journal name	Name of the journal
	Volume	Volume number
	Page numbers	Page numbers
	Doi	DOI number
	Url	DOI URL
Study characteristics	Study design	Type of study
	Data source	Primary or secondary
	Data collection	Data collection (quantitative, qualitative, mixed methods)
	Study location	Study site as defined in the study
	Country	Country name
	Ospar region	Name Ospar maritime region
	Coordinates	Latitude (WSG84; unit: Decimal). If many study locations, coordinates are for an intermediate point.
	Coordinates	Longitude (WSG84; unit: Decimal). If many study locations, coordinates are for an intermediate point.
Population	Type of coastal ecosystem affected	Type of coastal ecosystems - Seagrass, Salt marsh, Kelp forest
	Seagrass species	Seagrass species name
	Saltmarsh species	Saltmarsh species name
	Macroalgae species	Macroalgae (kelp, rockweed) species name
	Component(s) of ecosystem assessed	Biotope (substrate, water column) and/or biocenosis components (plants and associated organism (s))
Intervention	Type of sector	Sector of the human activity (e.g. Aquaculture, Coastal Infrastructure, Fishing etc.)
	Notes on sector	Comments on sector
	Type of activity	Human activity type (e.g. Fin-fish aquaculture, shellfish aquaculture, Marinas/port facilities, Artificial reef, Trawls and dredges, etc.)
	Notes on activity	Comments on activity
	Type of Pressure(s)	Pressure (e.g. Nutrient enrichment, Mechanical damage, Barrier to species movement, Selective extraction etc.
	Notes on pressure	Comments on pressures
Outcome	Target outcome	The outcome of the impact assessment: biodiversity or carbon.
	Impact assessment type	Either “assessed” (documented or quantified) or “inferred”.
	Level of Impact on Biodiversity	Level of impact on biodiversity (Genetic, Organism, Population, Community). Used “Not applicable” is the assessment is for carbon.
	Measurements biodiversity	Metrics used to measure impact on biodiversity (e.g. Species richness, Species Abundance, Diversity Indices etc.)
	Measurements carbon	Metrics used to measure impact on carbon (e.g. Net Primary production, Carbon export, Biomass, Sequestration rate etc.)
	Impact	Description of impact
	Direction of impact	Positive, negative, no impact, or unclear
	Citation justifying direction	Exact citation from article
	Start Date of Measurements	Year: YYYY
	End Date of Measurements	Year: YYYY
	Notes on impacts	Comments on impacts

### Study mapping and presentation

Metadata extracted from studies after full-text screening will be standardized for further analysis. To investigate and visualize patterns in the distribution and abundance of evidence on impacts of humans on blue carbon ecosystems, analyses targeting our primary and secondary research questions will be performed in R. For example, we will characterize the distribution evidence across various categories of blue carbon ecosystems, identifying associated human activities, and resulting pressure(s), and impacts, while also classifying the level of details for each of the factors identified. We will also summarize the extent of the evidence on biodiversity and/or carbon capacity, including if the impacts identified have been documented, quantified, or inferred. We will determine differences in the evidence base by factors, such as ecosystems type, human activity type, type of pressure and geographic location. Additionally, we will identify methodologies used to evaluate human impacts, the metrics assessed, and when evaluations were collected relative to biodiversity and/or carbon capacity. Our analysis will identify topics where sufficient evidence exists and gaps needing future empirical research. Heatmap plots and structured matrices will be used to identify evidence gaps and clusters among direct and indirect impact of human activities, studied populations and locations in the North-East Atlantic. Complementary visualization, including bar plots and geographic distribution maps, as well as tabular summaries will be used to comprehensively summaries the evidence base.

## Supplementary Information

Additional file 1. Benchmarking articles.

Additional file 2. ROSES for systematic map protocols checklist.

Additional file 3. Search strategy development and testing.

Additional file 4. Data extraction codebook.

## Data Availability

No datasets were generated or analysed during the current study.
