# Supplementary material for: What evidence exists on the impacts of human activities on biodiversity and carbon capacity in North-East Atlantic blue carbon ecosystems: a systematic map protocol
Source: Environ Evid. 2025 Dec 8;14:26. doi: 10.1186/s13750-025-00379-0 (PMC12715903; doi:10.1186/s13750-025-00379-0)
Supplement: Supplementary file 2 — Additional file 2. ROSES for systematic map protocols checklist. [file 13750_2025_379_MOESM2_ESM.pdf]

Item nb	Section / sub-section	Topic	Description	Further explanation	Checklist/Meta-data	Author response	Comments
1	Title	Title	The title must indicate that it is a systematic map protocol, and must indicate if it is an update/amendment: e.g. "A systematic map update protocol…".	The title should normally be the same or very similar to the review question.	Meta-data	What evidence exists on the impacts of human activities on biodiversity and carbon capacity in North-East Atlantic blue carbon ecosystems: a systematic map protocol.	
2	Type of review	Type of review	Select one of the following types of review: systematic map, systematic map update, systematic map amendment	See CEE Guidance on systematic mapping [1], and on amendments and updates [2]	Meta-data	systematic map	
3	Authors contacts	Authors contacts	The full names, institutional addresses, and email addresses for all authors must be provided.		Checklist	Yes	
4	Abstract	Structured summary	Abstract must not exceed 350 words and must include two sections 1) Background, the context and purpose of the review, including the review question; 2) Methods, how the review will be conducted and the outputs that are expected (specifically mention search strategy, inclusion criteria, critical appraisal, data extraction and synthesis).		Checklist	Yes	
5	Background	Background	Describe the rationale for the review in the context of what is already known. Protocol must indicate why this study was necessary and what it aims to contribute to the field.	A theory of change and/or conceptual model can be presented that links the intervention or exposure to the outcome.	Checklist	Yes	
6	Stakeholder engagement	Stakeholder engagement	The planned/actual role of stakeholders throughout the review process (e.g. in the formulation of the question) must be described and explained (using a broad definition of 'stakeholder', including e.g. researchers, funders and other decision-makers; see [3])		Checklist	Yes	Stakeholders other than researchers have not been consulted to elaborate the protocol
7	Objective of the review	Objective	Describe the primary question and secondary questions (when applicable).	The primary question is the main question of the review. Secondary questions are usually linked to sources of heterogeneity (effect modifiers).	Checklist	Yes	
8		Definitions of the question components	Break down and summarise question key elements e.g. population, intervention(s)/exposure(s), comparator(s), and outcome(s).	For other question types see [4,5]	Meta-data	Population:blue carbon ecosystems - seagrass meadows, saltmarshes and macroalgae (kelp and rockweed) in the North East Atlantic<br>Intervention:Human activities impacting biodiversity and/or carbon capacity blue carbon ecosystems<br>Outcome:There are no predefined outcomes. All outcomes will be potentially relevant as long as they are related to impacts from human activities on biodiversity and/or carbon capacity of the population studied.<br>Comparator: No comparator required	
	Methods						
9	Searches	Search strategy		Details regarding search strategy testing should be provided.	Checklist	Yes	
10		Search string	Provide Boolean-style full search string and state the platform for which the string is formatted (e.g. Web of Science format)		Meta-data	(TS=("coastal habitat" OR "intertidal habitat" OR "subtidal habitat" OR "coastal ecosystem" OR "blue carbon" OR "blue carbon ecosystem" OR "blue carbon habitat" OR marsh* OR saltmarsh* OR estuar* OR kelp OR "macroalga" OR seaweed OR rockweed* OR seagrass* OR "sea grass" OR eelgrass OR "coastal wetland") AND TS= (impact* OR effect OR effects OR "human disturbance*" OR "anthropogenic disturbance*" OR pressure* OR "human impact*" OR "human pressure*" OR "human activit*" OR "anthropogenic impact*" OR "anthropogenic pressure*") AND TS= (biodivers* OR "biological diversity" OR "biotic diversity" OR "species richness" OR "species abundance" OR "eveness" OR "species diversity" OR "communit* structure" OR "species composition*" OR "ecosystem* integrity" OR "macrofauna") AND TS= (biomass OR "carbon sequestrat*" OR "carbon stor*" OR "carbon stock*" OR "organic carbon" OR "carbon uptake*" OR "carbon sink*" OR "carbon capture*") AND AD=("Azores" OR "Basque" OR "Belgium" OR "Danmark" OR "Danish" OR "Denmark" OR "Dutch" OR "English" OR "England" OR "Faroe Island*" OR "Faroe*"OR "France" OR "French" OR "Germany" OR "German" OR "Greenland" OR "Greenlandic" OR "Holland" OR "Iceland" OR "Icelandic" OR "Ireland" OR "Irish" OR "Netherland" OR "Norway" OR "Norwegian" OR "Portugal" OR "Portuguese" OR "Scotland" OR "Scottish" OR "Spain" OR "Spanish" OR "Sweden" Or "Swedish" OR "United Kingdom" OR "UK" OR "Wales"))	
11		Languages – bibliographic databases	List languages to be used in bibliographic database searches.		Meta-data	English	
12		Languages – grey literature	List languages to be used in organizational websites searches and web-based search engines.		Meta-data	n/a	No grey literature search
13		Bibliographic databases	Provide the number of bibliographic databases to be searched.		Meta-data	2	
14		Web – based search engines	Provide the number of web – based search engines to be searched.		Meta-data	1	
15		Organisational websites	Provide the number of organisational websites to be searched.		Meta-data	0	
16		Estimating the comprehensiveness of the search	Describe the process by which the comprehensiveness of the search strategy was assessed (i.e. list of benchmark articles).		Checklist	Yes	
17		Search update	Describe any plans to update the searches during the conduct of the review.	Optional. A search update is good practice if original searches were performed more than two years prior to review completion.	Checklist	n/a	No search update to be done in this review
18	Article screening and study inclusion criteria	Screening strategy	Describe the methodology for screening articles/studies for relevance/eligibility.		Checklist	Yes	
19		Consistency checking	Describe clearly the process for checking consistency of decisions including the levels at which consistency checking will be undertaken and estimated proportion of articles/studies that will be screened and checked for consistency by two or more reviewers (e.g. Titles (10%), abstracts (10%), full text (10%)).		Checklist	Yes	
20		Inclusion criteria	Describe the inclusion criteria used to assess relevance of identified articles/studies. These must be broken down into the question key elements (e.g. relevant subject(s), intervention(s)/exposure(s), comparator(s), outcomes, study design(s)) and any other restrictions (e.g. date ranges or languages).		Checklist	Yes	
21		Reasons for exclusion	State that you will provide a list of articles excluded at full text with reasons for exclusion.		Checklist	Yes	
22	Critical appraisal	Critical appraisal strategy	Describe here the method you propose for critical appraisal of study validity (including assessment of individual studies and the evidence base as a whole).	Optional	Checklist	n/a	
23		Critical appraisal used in synthesis	Describe how the information from critical appraisal will be used in synthesis.	Optional	Checklist	n/a	
24		Consistency checking	Describe how repeatability of critical appraisal of study validity will be tested.	Optional	Checklist	n/a	
25	Data extraction	Meta-data extraction and coding strategy	Describe the method for meta-data extraction and coding for studies (potentially providing forms/data sheets (ideally piloted), list if variables to be extracted as meta-data and those that will be coded).		Checklist	Yes	
26	Data synthesis and presentation	Narrative synthesis strategy	Describe methods to be used for narratively synthesising the evidence base in the form of descriptive statistics, tables (including SM database) and figures.	Vote-counting (tallying of studies based on the direction or significance of their findings) must be avoided. May include a summary of the outputs of critical appraisal of the evidence base as a whole (if planned to be performed in SM).	Checklist	Yes	
27		Knowledge gap and cluster identification strategy	Describe the methods to be used to identify and/or prioritise key knowledge gaps (unrepresented or underrepresented subtopics that warrant further primary research) and knowledge clusters (well-represented subtopics that are amenable to full synthesis via systematic review).		Checklist	Yes	
28		Demonstrating procedural independence	Describe the role of systematic reviewers (who have also authored articles to be considered within the review) in decisions regarding inclusion or critical appraisal of their own work.	Reviewers who have authored articles to be considered within the review should be prevented from unduly influencing inclusion decisions, for example by delegating tasks appropriately.	Checklist	Yes	
29	Declarations	Competing interests	Describe of any financial or non-financial competing interests that the review authors may have.		Checklist	Yes	

References

[1] James, K.L., Randall, N.P. and Haddaway, N.R., 2016. A methodology for systematic mapping in environmental sciences. Environmental Evidence, 5(1), p.7.

[2] Bayliss, H.R., Haddaway, N.R., Eales, J., Frampton, G.K. and James, K.L., 2016. Updating and amending systematic reviews and systematic maps in environmental management. Environmental Evidence, 5(1), p.20.

[3] Haddaway, N.R., Kohl, C., da Silva, N.R., Schiemann, J., Spök, A., Stewart, R., Sweet, J.B. and Wilhelm, R., 2017. A framework for stakeholder engagement during systematic reviews and maps in environmental management. Environmental Evidence, 6(1), p.11.

[4] Collaboration for Environmental Evidence. 2018. Guidelines and Standards for Evidence synthesis in Environmental Management. Version 5.0. www.environmentalevidence.org/information-for-authors.

[5] Leeds Institute of Health Sciences. https://medhealth.leeds.ac.uk/info/639/information_specialists/1500/search_concept_tools. Accessed 12/11/2017.
