# Supplementary material for: What evidence exists on the impacts of human activities on biodiversity and carbon capacity in North-East Atlantic blue carbon ecosystems: a systematic map protocol
Source: Environ Evid. 2025 Dec 8;14:26. doi: 10.1186/s13750-025-00379-0 (PMC12715903; doi:10.1186/s13750-025-00379-0)
Supplement: Supplementary file 3 — Additional file 3. Search strategy development and testing. [file 13750_2025_379_MOESM3_ESM.pdf]

Search string development

Web od Science:

TS=Topic field

AD= Addresses

Blue carbon ecosystems and Human activities and Biodiversity OR Carbon capacity - (**TS=**(“coastal habitat” OR “intertidal habitat” OR “subtidal habitat” OR “coastal ecosystem” OR “blue carbon” OR “blue carbon ecosystem” OR “blue carbon habitat” OR marsh* OR saltmarsh* OR estuar* OR kelp OR “macroalga” OR seaweed OR rockweed* OR seagrass* OR “sea grass” OR eelgrass OR "coastal wetland”) **AND TS=**(impact* OR effect OR effects OR "human disturbance*" OR “anthropogenic disturbance*” OR pressure* OR "human impact*" OR "human pressure*" OR "human activit*" OR "anthropogenic impact*" OR "anthropogenic pressure*") **AND TS=((**biodivers* OR "biological diversity" OR “biotic diversity” OR “species richness” OR “species abundance” OR “eveness” OR “species diversity” OR “communit* structure” OR “species composition*” OR “ecosystem* integrity” OR “macrofauna”) OR **TS=** (biomass OR “carbon sequestrat*" OR "carbon stor*" OR "carbon stock*" OR "organic carbon" OR “carbon uptake*” OR “carbon sink*” OR “carbon capture*”)**) AND AD=**("Azores" OR “Basque” OR "Belgium" OR "Danmark" OR "Danish" OR "Denmark" OR “Dutch” OR "English" OR "England" OR "Faroe Island*" OR "Faroe*"OR "France" OR "French" OR "Germany" OR "German" OR "Greenland" OR "Greenlandic" OR "Holland" OR "Iceland" OR "Icelandic" OR "Ireland" OR "Irish" OR "Netherland" OR "Norway" OR "Norwegian" OR "Portugal" OR "Portuguese" OR "Scotland" OR "Scottish" OR "Spain" OR "Spanish" OR "Sweden" Or "Swedish" OR "United Kingdom" OR "UK" OR "Wales"))

Blue carbon ecosystems and Human activities and Biodiversity and Carbon capacity - (**TS=**(“coastal habitat” OR “intertidal habitat” OR “subtidal habitat” OR “coastal ecosystem” OR “blue carbon” OR “blue carbon ecosystem” OR “blue carbon habitat” OR marsh* OR saltmarsh* OR estuar* OR kelp OR “macroalga” OR seaweed OR rockweed* OR seagrass* OR “sea grass” OR eelgrass OR "coastal wetland”) **AND TS=** (impact* OR effect OR effects OR "human disturbance*" OR “anthropogenic disturbance*” OR pressure* OR "human impact*" OR "human pressure*" OR "human activit*" OR "anthropogenic impact*" OR "anthropogenic pressure*") **AND TS=** (biodivers* OR "biological diversity" OR “biotic diversity” OR “species richness” OR “species abundance” OR “eveness” OR “species diversity” OR “communit* structure” OR “species composition*” OR “ecosystem* integrity” OR “macrofauna”) **AND TS=** (biomass OR “carbon sequestrat*" OR "carbon stor*" OR "carbon stock*" OR "organic carbon" OR “carbon uptake*” OR “carbon sink*” OR “carbon capture*”) **AND AD=**("Azores" OR “Basque” OR "Belgium" OR "Danmark" OR "Danish" OR "Denmark" OR “Dutch” OR "English" OR "England" OR "Faroe Island*" OR "Faroe*"OR "France" OR "French" OR "Germany" OR "German" OR "Greenland" OR "Greenlandic" OR "Holland" OR "Iceland" OR "Icelandic" OR "Ireland" OR "Irish" OR "Netherland" OR "Norway" OR "Norwegian" OR "Portugal" OR "Portuguese" OR "Scotland" OR "Scottish" OR "Spain" OR "Spanish" OR "Sweden" Or "Swedish" OR "United Kingdom" OR "UK" OR "Wales"))

SCOPUS:

ABS: Abstract; KEY: Keywords: AFFIL: Affiliation; PUBYEAR: Publication Year; DOCTYPE: Document Type; “ar”: Article

Blue carbon ecosystems and Human activities and Biodiversity

TITLE-ABS-KEY ("coastal habitat" OR "intertidal habitat" OR "subtidal habitat" OR "coastal ecosystem" OR "blue carbon" OR "blue carbon ecosystem" OR "blue carbon habitat" OR marsh* OR saltmarsh* OR estuar* OR kelp OR macroalga* OR seaweed OR rockweed* OR seagrass* OR "sea grass" OR eelgrass OR "coastal wetland") AND

TITLE-ABS-KEY (impact* OR effect OR effects OR "human disturbanc*" OR pressure* OR "human impact*" OR "human pressure*" OR "human activit*" OR "anthropogenic impact*" OR "anthropogenic pressure*") AND

TITLE-ABS-KEY (biodivers* OR "biological diversity" OR "biotic diversity" OR "species richness" OR "species abundance" OR eveness OR "species diversity" OR "communit* structure" OR "species composition*" OR "ecosystem* integrity" OR macrofauna) AND

AFFIL (azores OR basque OR belgium OR danmark OR danish OR denmark OR dutch OR english OR england OR "Faroe Island*" OR faroe* OR france OR french OR germany OR german OR greenland OR greenlandic OR holland OR iceland OR icelandic OR ireland OR irish OR netherland OR norway OR norwegian OR portugal OR portuguese OR scotland OR scottish OR spain OR spanish OR sweden OR swedish OR "United Kingdom" OR uk OR wales)

Blue carbon ecosystems and Human activities and Carbon capacity

TITLE-ABS-KEY ("coastal habitat" OR "intertidal habitat" OR "subtidal habitat" OR "coastal ecosystem" OR "blue carbon" OR "blue carbon ecosystem" OR "blue carbon habitat" OR marsh* OR saltmarsh* OR estuar* OR kelp OR macroalga* OR seaweed OR rockweed* OR seagrass* OR "sea grass" OR eelgrass OR "coastal wetland") AND

TITLE-ABS-KEY (impact* OR effect OR effects OR "human disturbanc*" OR pressure* OR "human impact*" OR "human pressure*" OR "human activit*" OR "anthropogenic impact*" OR "anthropogenic pressure*") AND

TITLE-ABS-KEY (biomass OR "carbon sequestrat*" OR "carbon stor*" OR "carbon stock*" OR "organic carbon" OR "carbon uptake*" OR "carbon sink*" OR "carbon capture*") AND

AFFIL (azores OR basque OR belgium OR danmark OR danish OR denmark OR dutch OR english OR england OR "Faroe Island*" OR faroe* OR france OR french OR germany OR german OR greenland OR greenlandic OR holland OR iceland OR icelandic OR ireland OR irish OR netherland OR norway OR norwegian OR portugal OR portuguese OR scotland OR scottish OR spain OR spanish OR sweden OR swedish OR "United Kingdom" OR uk OR wales)

Blue carbon ecosystems and Human activities and Biodiversity and Carbon capacity

TITLE-ABS-KEY ("coastal habitat" OR "intertidal habitat" OR "subtidal habitat" OR "coastal ecosystem" OR "blue carbon" OR "blue carbon ecosystem" OR "blue carbon habitat" OR marsh* OR saltmarsh* OR estuar* OR kelp OR macroalga* OR seaweed OR rockweed* OR seagrass* OR "sea grass" OR eelgrass OR "coastal wetland") AND

TITLE-ABS-KEY (impact* OR effect OR effects OR "human disturbanc*" OR pressure* OR "human impact*" OR "human pressure*" OR "human activit*" OR "anthropogenic impact*" OR "anthropogenic pressure*") AND

TITLE-ABS-KEY (biodivers* OR "biological diversity" OR "biotic diversity" OR "species richness" OR "species abundance" OR eveness OR "species diversity" OR "communit* structure" OR "species composition*" OR "ecosystem* integrity" OR macrofauna) AND

TITLE-ABS-KEY (biomass OR "carbon sequestrat*" OR "carbon stor*" OR "carbon stock*" OR "organic carbon" OR "carbon uptake*" OR "carbon sink*" OR "carbon capture*") AND

AFFIL (azores OR basque OR belgium OR danmark OR danish OR denmark OR dutch OR english OR england OR "Faroe Island*" OR faroe* OR france OR french OR germany OR german OR greenland OR greenlandic OR holland OR iceland OR icelandic OR ireland OR irish OR netherland OR norway OR norwegian OR portugal OR portuguese OR scotland OR scottish OR spain OR spanish OR sweden OR swedish OR "United Kingdom" OR uk OR wales)

GOOGLE SCHOLAR

Blue carbon ecosystems and Human activities and Biodiversity and Carbon capacity –

(“coastal habitat” OR “intertidal habitat” OR “subtidal habitat” OR “coastal ecosystem” OR “blue carbon” OR “blue carbon ecosystem” OR “blue carbon habitat” OR marsh* OR saltmarsh* OR estuar* OR kelp OR “macroalga*” OR seaweed OR rockweed* OR seagrass* OR “sea grass” OR eelgrass OR "coastal wetland”) AND (impact* OR effect* OR "human disturbanc*" OR pressure* OR "human impact*" OR "human pressure*" OR "human activit*" OR "anthropogenic impact*" OR "anthropogenic pressure*") AND (biodivers* OR "biological diversity" OR “biotic diversity” OR “species richness” OR “species abundance” OR “eveness” OR “species diversity” OR “communit* structure” OR “species composition*” OR “ecosystem* integrity” OR “macrofauna”) AND (biomass OR “carbon sequestrat*" OR "carbon stor*" OR "carbon stock*" OR "organic carbon" OR “carbon uptake*” OR “carbon sink*” OR “carbon capture*”)
